# Development of a Telehealth-Enabled Portable Optical Endomicroscopy System with Targeted Peptides: A Preclinical Feasibility Study for Cervical Cancer Detection

**DOI:** 10.3390/cancers18081306

**Published:** 2026-04-20

**Authors:** Chanchai Thaijiam, Nitipon Navaitthiporn, Preeyarat Rithcharung, Nicholas Piyawattanametha, Shoji Komai, Supang Khondee, Wibool Piyawattanametha

**Affiliations:** 1 Faculty of Engineering, Srinakharinwirot University, Ongkharak Campus, Nakorn Nayok 26120, Thailand; chanchait@g.swu.ac.th; 2 Advanced Imaging Research Center, School of Engineering, King Mongkut’s Institute of Technology Ladkrabang, Ladkrabang, Bangkok 10520, Thailand; eark.navaitthiporn@gmail.com (N.N.); preeyaratnut@gmail.com (P.R.); 3 School of Engineering, Michigan State University, East Lansing, MI 48824, USA; piyawat1@msu.edu; 4 Department of Information Technology, Faculty of Technology, International Professional University of Technology, Tokyo 1-7-3 Nishi-Shinjuku, Shinjuku 160-0023, Tokyo, Japan; komai.shoji@iput.ac.jp; 5 School of Pharmaceutical Sciences, University of Phayao, Maeka, Phayao 56000, Thailand; supang.kh@up.ac.th; 6 Institute for Quantitative Health Science & Engineering, Michigan State University, East Lansing, MI 48824, USA

**Keywords:** cervical cancer, fluorescence imaging, optical endomicroscopy, targeted peptide, telehealth

## Abstract

Background/Objectives: We developed a telehealth-enabled fiber-bundle endomicroscopy platform and evaluated its preclinical feasibility for targeted fluorescence imaging in cervical cancer models. Methods: The platform integrates a portable fiber-bundle endomicroscopy (FBE) system, fluorescein isothiocyanate (FITC)-labeled candidate peptides, and a secure web-based telehealth platform for remote consultation. The FBE probe achieved a field of view of 1,700 µm and a lateral resolution of 4 µm, enabling cellular-level fluorescence imaging in a compact, portable format. Four FITC-labeled peptides (SHS1*, SHS2*, FPP*, and CRL*) were evaluated in A549, SiHa, and CaSki cell lines. Ex vivo testing was performed on commercial cervical tissue-array samples. The telehealth platform was assessed for secure medical-image/video transmission and end-to-end latency in a simulated remote-consultation setting. Results: Among the tested probes, FPP*-FITC and CRL*-FITC showed higher fluorescence-positive fractions in the p16-overexpressing cervical cancer cell lines than in the A549 comparator line, with the strongest signals observed in CaSki cells. In ex vivo testing, CRL*-FITC generated higher fluorescence intensity in malignant cervical tissue-array samples than in non-malignant comparator tissues, with a reported 4.6- to 7.4-fold difference in mean signal intensity (*p* < 0.001). The telehealth platform supported the secure transmission of medical images and video and demonstrated an end-to-end latency of <500 ms in a simulated remote consultation setting. Conclusions: These results support the technical and preclinical feasibility of integrating targeted fluorescence imaging, portable fiber-bundle endomicroscopy, and telehealth into a single platform. This study should therefore be interpreted as a preclinical feasibility study evaluating optical, molecular, and telehealth integration, rather than as a clinically validated cervical cancer screening test.

## Simple Summary

Cervical cancer remains a major health problem, especially in settings where access to specialists and follow-up care is limited. In this study, we developed a portable fiber-bundle endomicroscopy platform combined with fluorescein-labeled candidate peptides and a secure telehealth interface for remote image review. The platform achieved cellular-level fluorescence imaging and was evaluated in p16-overexpressing cervical cancer cell lines, commercial ex vivo tissue array samples, and a simulated remote consultation workflow. Two peptides, FPP*-FITC and CRL*-FITC, showed higher fluorescence signals in p16-overexpressing cervical cancer models than in the comparator cell line, and CRL*-FITC produced higher fluorescence in malignant than in non-malignant ex vivo tissue-array samples. In addition, the telehealth platform supported secure image transfer and low-latency remote communication. These findings support the preclinical feasibility of combining targeted fluorescence imaging with a portable endomicroscope and telehealth. Further mechanistic and clinical studies will be required to determine diagnostic performance and clinical utility in future cervical imaging and screening workflows.

## 1. Introduction

Cervical cancer remains a major public-health problem in Thailand and other settings where access to prevention, screening, and follow-up care is uneven [1,2]. In Thailand, a large population remains at risk, and cervical cancer continues to contribute substantially to cancer-related morbidity and mortality among women [1,2]. Cervical carcinogenesis is now well established as strongly associated with persistent infection with high-risk human papillomavirus (hrHPV), rather than with an unknown cause [2]. Therefore, the continuing burden of disease is better understood as reflecting uneven access to vaccination, screening, diagnostic confirmation, and timely treatment, particularly in some rural and resource-constrained settings [1,2].

Current cervical-cancer screening pathways include cervical cytology, primary hrHPV testing, and cotesting, with the exact strategy depending on age, risk group, and local programmatic context [3,4,5,6]. American College of Obstetricians and Gynecologists (ACOG) and the United States Preventive Services Task Force (USPSTF) both recognize three screening options for average-risk individuals aged 30–65 years: cytology alone, primary hrHPV testing alone, or cotesting [3,4]. In Thailand, Royal Thai College of Obstetricians and Gynaecologists (RTCOG) guidance also reflects the role of hrHPV-based screening and follow-up pathways, including primary HPV-based approaches [5,6]. Accordingly, it is more accurate to describe cotesting as one accepted screening option, rather than as the single international standard [3,4,5,6].

In practice, each screening modality has important limitations. Cytology depends on adequate specimen collection, slide preparation, and expert interpretation, and its performance may be reduced by sampling error or limited laboratory capacity [7,8]. High-risk HPV testing can improve sensitivity for detecting clinically relevant diseases. However, positive results still require appropriate triage and follow-up because HPV infection is common and not all infections progress to precancer or cancer [3,4,6,9,10]. As a result, abnormal screening results commonly lead to colposcopic evaluation and, when indicated, biopsy for histopathologic confirmation before treatment planning [4,6]. Thus, even with contemporary screening strategies, the overall pathway can still require multiple steps and repeated visits [6,11].

Given the technical and workflow limitations described above, there remains a need for approaches that provide more immediate, lesion-directed information at the point of care while reducing dependence on centralized laboratory infrastructure and repeated follow-up visits. In low-resource or geographically remote settings, these challenges are compounded by limited access to specialists and by the risk of patient loss to follow-up between screening, confirmatory assessment, and treatment planning [11]. Telehealth may help address part of this gap by enabling remote review of images and clinical findings. Still, its usefulness depends on the availability of portable, sufficiently high-resolution image acquisition tools compatible with secure digital transmission [12,13].

In this context, fiber-bundle endomicroscopy (FBE) is of interest because it can provide compact, portable, cellular-level fluorescence imaging using a relatively simple optical architecture [14,15,16]. However, optical imaging alone does not provide molecular contrast [16]. To improve lesion-associated fluorescence contrast, this work explores fluorescein-labeled candidate peptides derived from prior p16-binding peptide development. p16 is widely used as a biomarker in HPV-related cervical neoplasia [17]. Our earlier phage-display study identified candidate p16-binding peptides that preferentially interact in p16-overexpressing models and competitively block anti-p16 antibody binding, supporting their further investigation as molecular imaging probes [18]. At the same time, because p16 is an intracellular cell-cycle regulator and the precise interaction mechanism of the current *FITC*-labeled synthetic peptides remains unresolved, the present study does not assume that these probes establish cell-surface specificity or clinical diagnostic specificity; rather, they are evaluated here as candidate fluorescence probes in preclinical models [18,19,20].

Based on this rationale, we developed an integrated platform combining a portable FBE, *FITC*-labeled candidate peptides, and a secure telehealth interface for remote consultation. The purpose of this study was to evaluate the technical and preclinical feasibility of this combined optical, molecular, and digital workflow. Specifically, we assessed the imaging performance of the FBE platform, examined peptide-associated fluorescence in cervical cancer cell lines and archived ex vivo tissue-array specimens, and tested secure image transmission and low-latency remote communication within a simulated telehealth workflow.

Accordingly, the present work should be interpreted as a preclinical feasibility study evaluating the integration of optical, molecular, and telehealth modalities, rather than as a clinically validated cervical cancer screening test. Section 2 reviews the related literature in the three domains most relevant to the present study—fiber-bundle endomicroscopy, peptide-based molecular contrast, and telehealth systems. Section 3 describes the study methodology, including peptide preparation, fluorescence imaging, ex vivo tissue-array analysis, image processing, and statistical analysis. Section 4 presents the experimental results for the FBE platform, peptide-associated fluorescence in cervical cancer models, ex vivo tissue-array imaging, and telehealth system performance. Section 5 discusses the findings in the context of preclinical feasibility, biological interpretation, and translational limitations, and the Study Limitations Section summarizes the main study limitations. Finally, Section 6 provides the conclusions and outlines directions for future work.

## 2. Related Work

The present study brings together three technical domains: portable fiber-bundle endomicroscopy platform, peptide-based fluorescence targeting, and telehealth-enabled remote consultation. The related work is therefore reviewed according to these three components. First, FBE-based systems have been investigated for high-resolution, cellular-level imaging in compact optical formats. Second, molecular probes such as targeted fluorescent peptides have been explored to enhance biologically relevant contrast beyond morphology alone. Third, telehealth platforms provide the digital infrastructure for secure image sharing, communication, and specialist input across geographically separated care settings. On this basis, the following sections summarize prior work in each area and establish the background for the current integrated platform, which should be interpreted as a preclinical feasibility study rather than a clinically validated screening system.

### 2.1. Optical FBE Technology

FBE forms the optical imaging foundation of the proposed platform because it enables compact, cellular-level fluorescence imaging in a potentially portable format suitable for point-of-care use. Many biological and clinical imaging applications require visualization of tissue morphology at high spatial resolution, and several intravital techniques, including confocal, multiphoton, and second-harmonic microscopy, have demonstrated strong performance for in situ imaging. However, these systems generally require complex optical architectures, scanning mechanisms, and high-cost light sources, which can limit their practicality outside specialized laboratories or tertiary-care centers [16,21].

In contrast, optical FBE systems offer a simpler imaging architecture in which an ordered fiber bundle relays excitation light to the tissue and returns emitted or reflected light for image formation. Prior studies have shown that FBE can be configured as a wide-field epi-fluorescence microscope capable of cellular-level imaging in living tissues and fluorescently labeled specimens [22,23]. This makes FBE attractive as a platform technology for portable fluorescence imaging, especially when rapid image acquisition and broad field coverage are desired. At the same time, the technique has important limitations. Because image formation depends on the discrete cores within the fiber bundle, spatial resolution is constrained by fiber-core diameter and spacing, which introduces a characteristic honeycomb sampling pattern and can reduce image continuity. In addition, wide-field FBE lacks optical sectioning, so out-of-plane fluorescence and subsurface background may contribute to the detected signal.

To address these limitations, prior work has explored computational post-processing approaches to improve image quality and apparent resolution from fiber-bundle data, including motion-based frame combination and interpolation strategies [14,24]. These advances support the use of FBE as a practical optical basis for the present study. In the context of this work, FBE is not presented as a clinically validated screening modality in itself, but rather as the imaging component of an integrated preclinical feasibility platform designed to be combined with targeted fluorescence probes and remote consultation capabilities.

### 2.2. Peptide-Based Molecular Contrast

Optical morphology alone may be insufficient to distinguish abnormal from non-abnormal tissue with adequate biological specificity. For this reason, the second component of the proposed platform is peptide-based molecular contrast, in which fluorescently labeled candidate peptides are used to generate a lesion-associated optical signal. Molecular approaches to cancer detection frequently rely on selective interactions between a probe and a biological target, often a biomarker associated with malignant or premalignant transformation. Antibodies have been widely used for this purpose, but peptide-based probes offer several practical advantages, including small size, synthetic accessibility, ease of chemical modification, and compatibility with fluorescent labeling.

In this study, p16 was selected as the biological target of interest because it is a well-established biomarker associated with HPV-related cervical neoplasia and is commonly used in pathology to support lesion assessment [17]. Our prior phage-display study identified candidate p16-binding peptides and demonstrated preferential interaction in p16-overexpressing models, including competitive blocking of monoclonal antibody binding for a leading candidate [18]. In addition, one candidate probe (CRL*) was adapted from a previously reported phage-display peptide that promoted targeting and internalization in HPV-transformed cervical cancer cell lines [19]. In our prior p16-binding study, candidate phage-displayed peptides showed higher interaction with p16-overexpressing CaSki cells than with control cells, supporting the plausibility of p16-related targeting in model systems [18]. Together, these prior studies support further investigation of candidate peptides as molecular probes for fluorescence imaging. In the present manuscript, these peptides are considered candidate fluorescence probes for preclinical evaluation, rather than fully validated targeting ligands. This distinction is important because p16 is an intracellular biomarker, and the current study does not directly establish whether the *FITC*-labeled synthetic peptides bind at the cell surface, enter cells before interacting with intracellular targets, or partly accumulate through nonspecific uptake [18,19,20]. Accordingly, the peptide-related literature is relevant here not as proof of clinical specificity, but as the molecular rationale for testing whether targeted fluorescence contrast can complement the structural imaging capability of FBE.

Fluorescence labeling provides the signal-generation mechanism that links peptide interaction to optical visualization. Fluorescent molecules absorb excitation light and then emit photons at longer wavelengths after energy relaxation, allowing the emitted fluorescence to be separated from the excitation source. Fluorescein and its derivative, *FITC*, are widely used as tracers in biological imaging because they can be conjugated to molecular probes and visualized with standard fluorescence optics [21]. In this work, *FITC* labeling was used to convert the candidate peptides into optically detectable probes that could be visualized with the FBE platform. Thus, the role of peptide-based molecular contrast in the present study is to provide preferential fluorescence enrichment in p16-overexpressing cervical cancer models, rather than to claim definitive molecular specificity or established *in vivo* diagnostic performance.

### 2.3. Telehealth Infrastructure for Remote Consultation

The third component of the proposed platform is a telehealth infrastructure for remote consultation. The motivation for including this component is practical rather than purely technical: in many settings, especially in rural or resource-limited regions, access to specialists who can interpret cervical imaging findings may be limited, and delays between examinations, referrals, and follow-ups can contribute to fragmented care. Digital transmission of imaging data and communication between local providers and remote experts may help address part of this gap, provided that the system supports the timely, secure, and reliable exchange of information.

Telemedicine has become an increasingly important part of healthcare delivery, with applications ranging from routine outpatient consultation to emergency care and distributed specialist support [12,25]. In Thailand, telemedicine and related health information technologies are particularly relevant for extending clinical expertise across geographically dispersed facilities and improving continuity of care [26,27]. At the same time, successful implementation depends on more than just video communication. Interoperability across heterogeneous systems, protection of patient privacy, secure transmission of sensitive data, and dependable system performance all remain major requirements for broader deployment [13,25,27].

Within the present study, telehealth is included as the digital infrastructure that connects image acquisition to remote expert review. Its role is to support the secure transfer of fluorescence images, video, and related clinical information within a simulated consultation workflow. However, this component should be interpreted as a technical feasibility layer of the integrated platform rather than as evidence of clinically validated telemedicine performance. No interobserver agreement study, remote diagnostic concordance analysis, or clinical usability assessment was performed in the present work. Therefore, the telehealth literature reviewed here positions the system as a realistic remote-consultation architecture for future translational development, rather than as a proven clinical deployment model.

## 3. Materials and Methods

This study evaluated the preclinical feasibility of an integrated platform combining FBE, *FITC*-labeled candidate peptides, and a telehealth interface for remote consultation. The methodological framework was designed to assess three components of the proposed platform: (i) optical performance of the FBE platform for cellular-level fluorescence imaging, (ii) peptide-associated fluorescence in p16-overexpressing cervical cancer models, and (iii) technical feasibility of secure image transmission and remote consultation within a simulated telehealth workflow. On this basis, the study focused on engineering integration and preclinical validation rather than on clinical screening performance.

**Figure 1:**
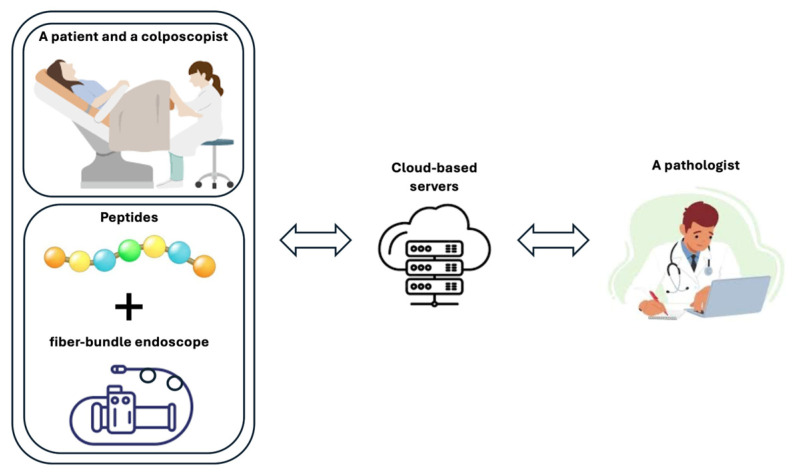
Conceptual workflow of the proposed preclinical FBE–peptide–telehealth platform.

This study was designed as an experimental preclinical feasibility investigation, and its conceptual workflow is illustrated in Figure 1. The workflow summarizes the integration of three components: candidate peptide preparation, cellular-level fluorescence imaging using an FBE, and digital transmission of imaging information through a telehealth interface. The peptide component was included to provide fluorescence contrast in p16-overexpressing cervical cancer models, while the optical component was developed to capture high-resolution fluorescence images in a portable format. These components were then integrated with a remote-consultation framework to evaluate secure data handling and low-latency communication in a simulated telehealth setting. Accordingly, the workflow should be interpreted as a framework for optical, molecular, and telehealth integration, rather than as a clinically validated cervical cancer screening pathway.

**Figure 2:**
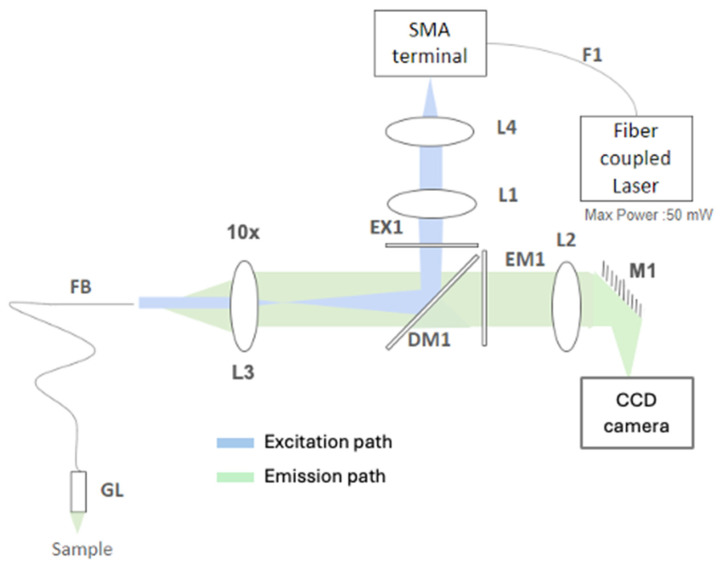
Optical setup of the fiber-bundle endomicroscopy system used in this study.

The FBE imaging system used in this study (Figure 2) employs a 488 nm fiber-coupled laser source (CNI Optoelectronics Technology Co., Ltd., Changchun, China, MBL-SF-491-50 mW). To ensure quantitative imaging, the laser is driven by a regulated constant-current supply (CNI, PSU-III-LED), which provides stable, adjustable output and minimizes intensity drift during long acquisitions. This stability is critical because fluctuations in excitation directly propagate into variability in the fluorescence signal. During initial coupling and periodic service, a 5× beam expander (CNI, 5×) is used upstream of the coupling optics to optimize beam diameter and mode fill, improving coupling efficiency and reducing sensitivity to pointing. The laser is delivered to the instrument through a 200 µm, 0.2 numerical aperture (NA) multimode fiber patch cord (F1, Thorlabs, Newton, NJ, USA, M92L02; SMA). At the SMA terminal, the excitation path proceeds through two Ø1″ lenses used for collimation and beam sizing—L4 (Thorlabs, Inc., ACL2520U, f = 20.1 mm) and L1 (Thorlabs, Inc., AC254-100-B, f = 100 mm). The beam is spectrally cleaned by the excitation band-pass filter EX1 (Semrock, Inc./IDEX, Rochester, NY, USA, FF02-482/18-25; >93% T from ~473–491 nm, centered at 482 nm) and then reflected toward the sample arm by the 488 nm dichroic mirror DM1 (Semrock, Inc./IDEX, Di03-R488-t1).

A 10× objective (L3, Olympus Corporation, Tokyo, Japan, MPLN10×, NA 0.25, working distance (WD) 10.6 mm) images the conditioned beam into the proximal face of the coherent imaging fiber bundle FB (Fujikura, Tokyo, Japan, FIGH-30-850N; ~30,000 cores, 650 µm active diameter). At the distal end, a gradient index (GRIN) relay GL (GRINTECH GmbH, Jena, Germany, GT-IFRL-100-005-50-CC; Ø1.0 mm, WD 5 mm, NA 0.5) focuses excitation onto the cervical epithelium, boosting effective NA at the tissue for better resolution and collection efficiency. Fluorescence generated in the tissue retraces the collection arm GL → FB → L3, then passes through DM1 (which transmits green emission while rejecting residual 488 nm light). The emission is further cleaned by EM1 (Semrock, Inc./IDEX, FF02-524/24-25; centered at 524 nm, matched to *FITC* emission ~520 nm). A 150 mm achromatic doublet L2 (Thorlabs, Inc., AC254-150-A) forms the imaging relay to the detector, with a plane dielectric mirror M1 (Thorlabs, Inc., NB1-J11; high-R 520–647 nm) folding the path to maintain a compact footprint.

Images are recorded on a high-sensitivity charge-coupled device (CCD) camera (FLIR Grasshopper2, Wilsonville, OR, USA), enabling cellular-scale visualization with high frame rates. This comprehensive optical architecture ensures a stable current drive (PSU-III-LED), controlled pre-coupling via the 5× expander, fiber delivery (F1), and precise free-space conditioning (L4, L1, EX1). The integration of efficient separation (DM1), high-NA objective coupling (L3), coherent transport (FB), and distal focusing (GL) maintains constant excitation intensity. Ultimately, the stringent emission filter (EM1) before the L2-M1-camera train enables robust pump and excitation light rejection, supporting repeatable, low-noise fluorescence imaging under the tested conditions. Table 1 lists the optical components and key specifications. Figure 2 summarizes the excitation path (laser→ F1 → L4 → L1 → EX1 → DM1 → L3 → FB → GL) and the fluorescence path (GL → FB → L3 → DM1 → EM1 → L2 → M1 → CCD).

**Figure 3:**
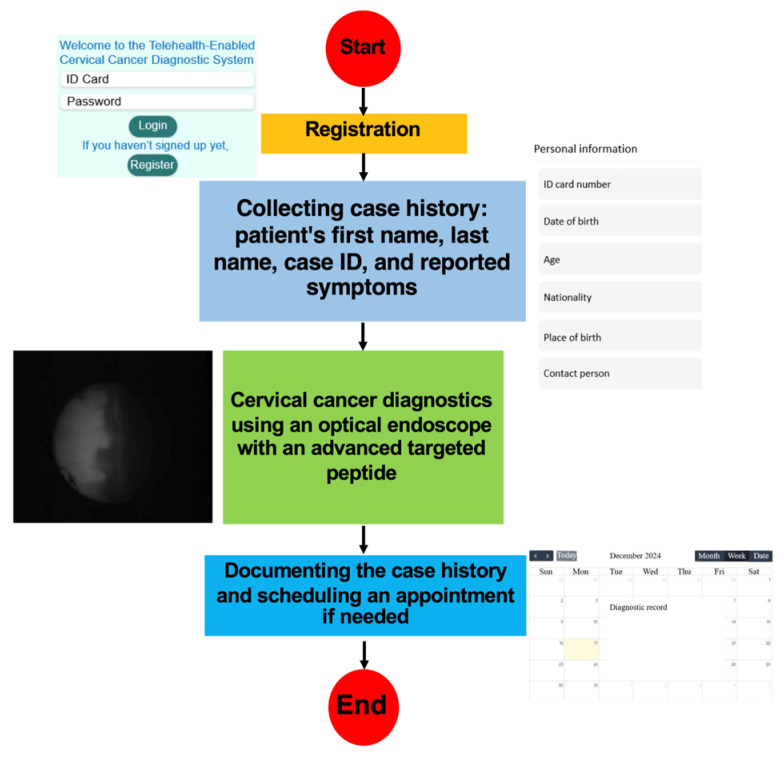
Workflow diagram of the proposed telehealth-supported remote-consultation process.

**Figure 4:**
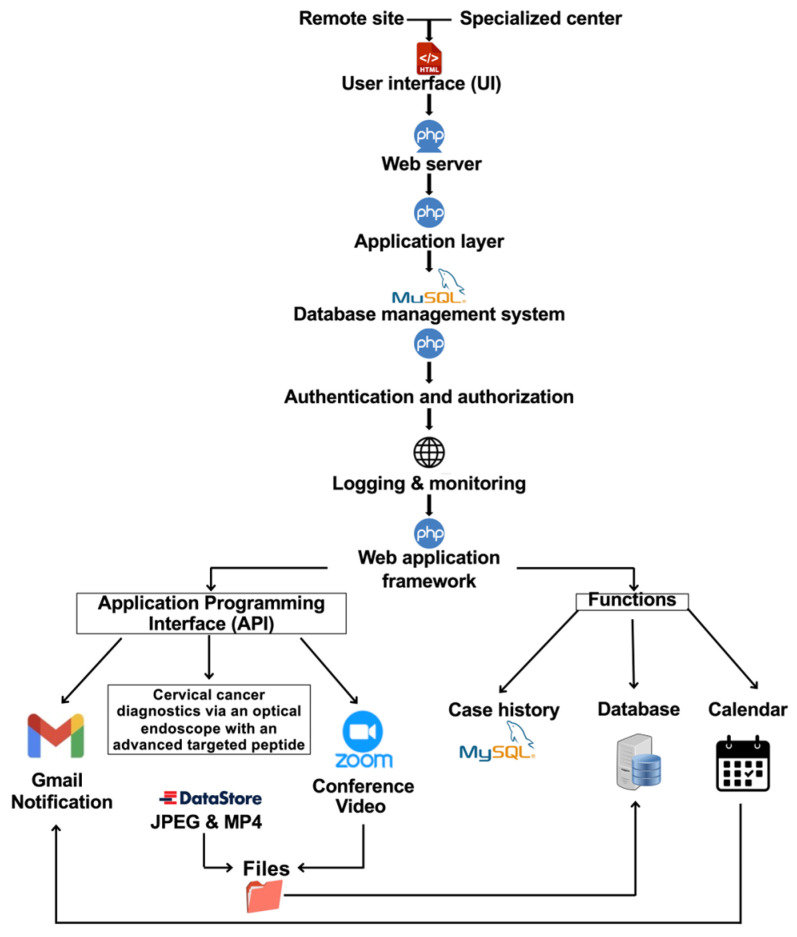
Software architecture of the proposed telehealth platform for remote consultation.

The operational workflow of the telehealth component is shown in Figure 3. In the proposed architecture, case identification (ID) and imaging data are entered at a remote site, transmitted to the consultation platform, reviewed by an authorized specialist, and stored for subsequent documentation and case management. The workflow is intended to illustrate how image acquisition, data entry, remote review, and follow-up coordination can be integrated within a single digital environment. In the present study, this workflow was evaluated as a technical consultation model rather than a clinically deployed screening pathway. The software architecture of the proposed telehealth platform is shown in Figure 4. The user interface (UI) supports entry and review of text, images, and video from remote and specialist sites through a web-based environment. The platform was designed to support authentication, secure data transmission, storage, retrieval, and communication within the simulated remote-consultation workflow evaluated in this study.

The user interface (UI) uses Hypertext Markup Language (HTML) to present the data-entry forms for these sites. Submitted data is transmitted to the web server via a Hypertext Preprocessor (PHP), which serves as an intermediary between users and the database. Once received by the server, the data moves to the application layer for processing. PHP is used to validate data and verify the host, username, and password to establish a secure connection to the Structured Query Language (SQL) database, which manages data storage and retrieval. After submission, user data undergoes authentication and authorization checks to verify identity and access privileges. For secure access to the remote site and specialized center data, a one-time password (OTP) is sent via email. This process leverages PHP Mailer and JavaScript to connect the user-input platform to the email system. Logging and monitoring functions are also incorporated for auditing and system administration. The application is built on a web application framework that supports external data validation via an application programming interface (API). Validation results are displayed via the UI, which interacts directly with the SQL database to enable accurate data retrieval and editing.

Furthermore, the framework integrates conferencing capabilities, allowing users to join Zoom meetings or access optical endomicroscopy applications via UI links. Accessing the endomicroscopy application involves preparing a dataset of categorized images and videos, organized in an image datastore with labels derived from folder names. The processed visual data is then stored in the database for diagnostic purposes, with results presented in text, image, and video formats to assist in diagnosis. Finally, both endoscopists and physicians can review patient case histories and manage appointment schedules via the system’s calendar.

To assess the technical feasibility of remote image review, an end-to-end latency test was designed for the telehealth platform. A simulated remote consultation was established over a standard 4 G/5 G cellular network, connecting the FLIR Grasshopper2-equipped FBE system at the remote site to the specialist center’s digital viewer. Latency was quantified by timestamping frame generation at the source and measuring the elapsed time until the corresponding frame was rendered on the remote display. Together, these software elements were implemented to assess the feasibility of secure image handling, communication, and case management within a telehealth-oriented research workflow, rather than to establish a clinically validated deployment model.

### 3.1. Peptide Source and Rationale

The candidate peptides evaluated in this study were selected from prior phage-display-derived peptide studies relevant to cervical cancer targeting, including our previous p16-binding peptide development work [18] and a previously reported HPV-transformed-cell targeting peptide study [19]. In these earlier studies, candidate peptides were identified through biopanning and were subsequently evaluated in p16-overexpressing cancer models, including competitive reduction in anti-p16 antibody staining for a leading candidate. The peptide constructs carried forward into the present study correspond to selected candidates from that prior work. They are summarized in Table 2, which includes their peptide identifiers, parent candidates from the previous study, amino acid sequences, and C-terminal labeling format. In the present work, synthetic *FITC*-labeled versions of these selected peptide sequences were prepared with a C-terminal GGGSK-*FITC* linker and used as fluorescence probes for preclinical evaluation with the FBE platform. These probes were evaluated as candidate molecular contrast agents in p16-overexpressing cervical cancer models to determine whether they could provide peptide-associated fluorescence enrichment under the conditions tested. However, they should not be interpreted as fully validated targeting ligands, and the present study does not assume that the observed signal establishes membrane-specific binding or clinical diagnostic specificity.

Following selection and synthesis of these candidate *FITC*-labeled peptide constructs, peptide-associated fluorescence was next assessed by flow cytometry in relevant cell models to compare their relative enrichment in p16-overexpressing cervical cancer cell lines under standardized conditions.

### 3.2. Flow-Cytometric Assessment of Peptide-Associated Fluorescence

A549 (ATCC CCL-185), SiHa (ATCC HTB-35), and CaSki (ATCC *CRL-1550*) cells were cultured to 70–80% confluence, harvested, and resuspended in phosphate-buffered saline (PBS). To reduce nonspecific interactions, cells were incubated with 0.5% bovine serum albumin (BSA) in PBS for 30 min at 4 °C, followed by PBS wash. Cells were subsequently incubated with 20 μM FITC-labeled peptide solution (SHS1*-FITC, SHS2*-FITC, FPP*-FITC, or CRL*-FITC) at 37 °C for 10 min, washed with PBS, fixed with 4% paraformaldehyde for 5 min, and washed again prior to analysis. Fluorescence-positive cell fractions were quantified using a Cytomics™ FC500 flow cytometer (Beckman Coulter, Brea, CA, USA), and the data were analyzed using FlowJo Vx software (version 10.10.0). Each peptide–cell line condition was evaluated in three independent experiments, and the reported values represent the mean ± standard deviation (SD) of the fluorescence-positive cell fraction across experiments. In the context of this assay, the reported percentages represent the fraction of cells classified as fluorescence-positive after peptide incubation, based on the applied flow-cytometry gating criteria. Because peptide incubation was performed before fixation, the measured signal should be interpreted as peptide-associated fluorescence after short incubation, which may reflect surface association and/or cellular uptake, rather than definitive membrane-specific binding.

### 3.3. Fluorescence Imaging of CRL*-FITC in Cell Samples and Ex Vivo Tissue-Array Samples

For comparative fluorescence imaging, SiHa cells were incubated with 50 µM CRL*-FITC and imaged using both the FBE and a standard fluorescence microscope under the acquisition conditions described in Section 4.4. A commercial human tissue array (TissueArray.com, Derwood, MD, USA, T103b) consisting of 30 spots (16 malignant cervical cores, 8 normal cervical cores, 2 adrenal tissue cores, and 4 blank positions) was used for exploratory ex vivo imaging. The CRL*-FITC was applied at a concentration of 25 µM. To provide exploratory signal-contrast data, four representative cores (A9, B9, C9, and C10) were selected for quantitative analysis. The selection criterion was to provide representative, high-quality examples of the distinct tissue conditions present on the array (malignant, non-malignant, and non-cervical control) to illustrate optical contrast. Representative fluorescence images of these selected cores were acquired using the FBE under identical acquisition settings, and fluorescence-intensity histograms were generated from the recorded images. In the present study, the reported fluorescence values for selected tissue-array spots were derived from image-based pixel-intensity distributions. Normal cervical tissue was treated as the primary biological reference, whereas adrenal tissue was included only as an additional non-cervical exploratory control available on the array. Because the tissue-array analysis was based on archived commercial specimens and image-derived intensity distributions, the resulting data should be interpreted as exploratory optical-contrast measurements rather than definitive estimates of clinical performance.

### 3.4. Image Analysis

Fluorescence images were analyzed by extracting grayscale intensity information from the acquired image frames. For each of the selected representative tissue-array spots, one single tissue-containing region of interest (ROI) encompassing the entire valid image area (>1.4 million pixels) was defined while excluding blank background regions. Grayscale pixel-intensity histograms were then extracted from each ROI, and mean ± SD values were calculated from the corresponding pixel-intensity distributions. To evaluate exploratory optical contrast, statistical comparisons were performed at the pooled pixel-level (comparing the pixel-intensity distributions directly) rather than at the spot-level. Fold-change values were calculated relative to the normal cervical reference sample. These image-derived measurements were used to compare fluorescence contrast between malignant and comparator tissues under identical imaging conditions. Because fluorescence in the tissue-array images was spatially heterogeneous and punctate, the resulting pixel-intensity distributions were expected to be non-normal and right-skewed.

### 3.5. Statistical Analysis

Quantitative data are reported as mean ± SD. For the flow-cytometry assay, the reported positive-cell fractions summarize the measured fluorescence-positive percentages obtained under each experimental condition. For the tissue-array experiment, the reported mean ± SD values were derived from grayscale pixel-intensity distributions extracted from ROIs of representative array spots. Because the fluorescence signal was spatially punctate against a dark background, these pixel-intensity distributions were highly right-skewed. Accordingly, standard deviations may exceed the mean and are reported here to illustrate signal heterogeneity within the analyzed ROIs rather than normally distributed biological variance. Statistical comparison of tissue-image intensity distributions was performed using a Mann–Whitney U test, and a *p*-value < 0.05 was considered statistically significant. Because the present study was designed as a preclinical feasibility investigation and some experiments relied on representative archived specimens, the statistical analyses should be interpreted as descriptive or exploratory rather than as estimates of clinical diagnostic performance.

All procedures were reviewed and approved by the Institutional Review Board of King Mongkut’s Institute of Technology Ladkrabang (Protocol: EC-KMITL 64 034), which determined that individual patient consent was not required because the study used anonymized commercial tissue samples and established cell lines.

## 4. Results

### 4.1. FBE Experiments

**Figure 5:**
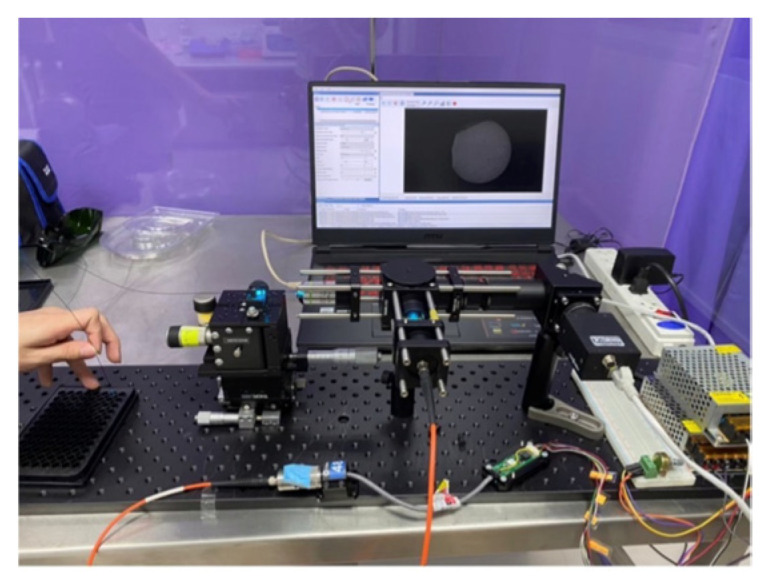
Optical setup of the FBE system.

**Figure 6:**
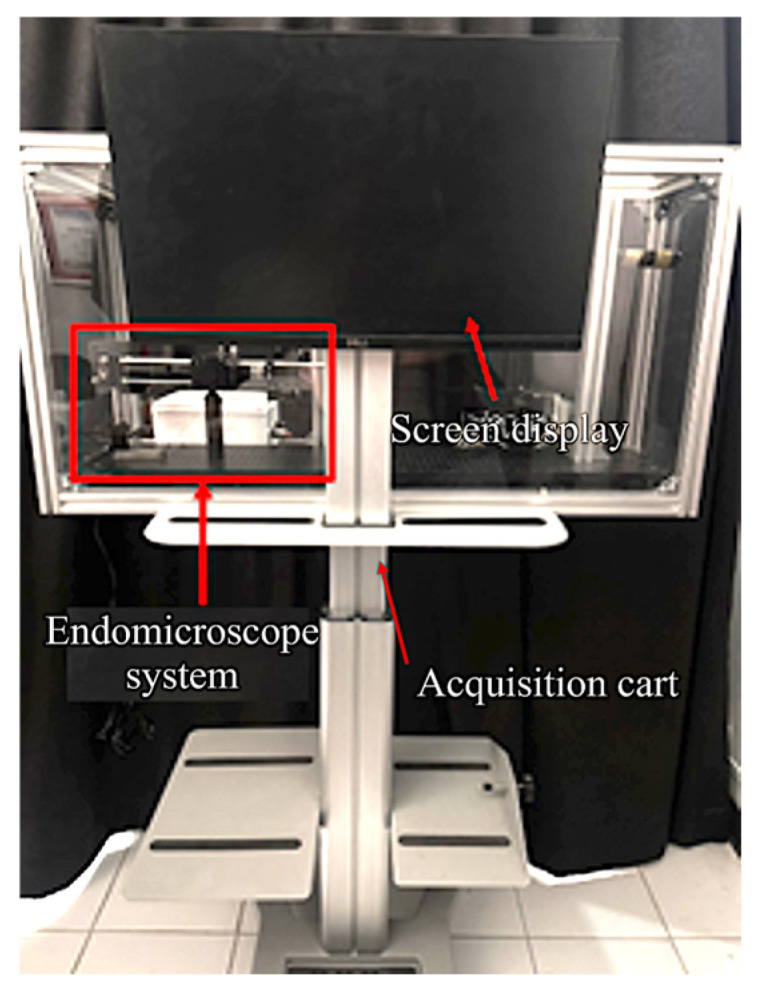
Optical imaging cart for the FBE system.

The optical setup of the FBE system and the image acquisition cart are shown in Figure 5 and Figure 6, respectively. The setup includes the FBE, associated optical components, and an imaging camera for fluorescence acquisition. Under the tested configuration, the system achieved a field of view (FOV) of 1700 μm in diameter and a lateral resolution of 4 μm, supporting cellular-level fluorescence imaging. The image acquisition cart was designed to house the power supplies and data-acquisition units required for transportable operation in laboratory and potential point-of-care environments. These results demonstrate the platform’s technical imaging performance and portability, but do not, by themselves, establish clinical screening accuracy or diagnostic performance.

**Figure 7:**
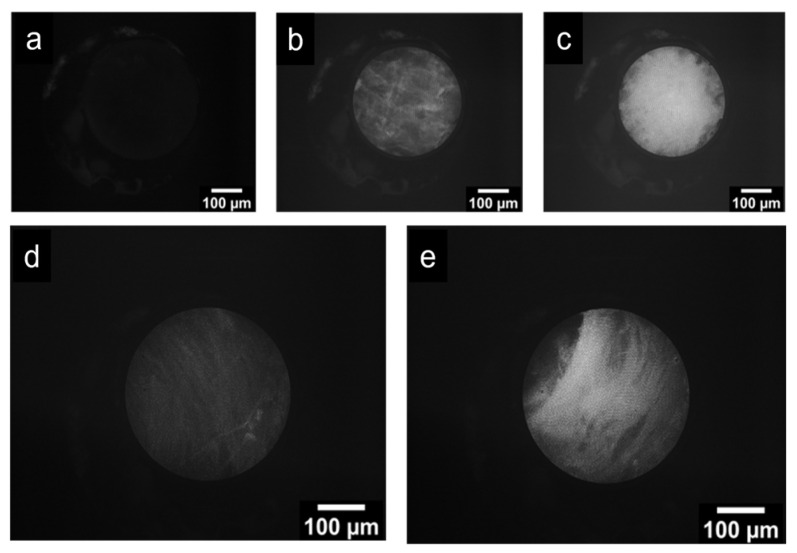
Fluorescence imaging of tissue samples stained with *FITC* at varying concentrations using the FBE. (**a**) Control sample, (**b**) 0.01% *w*/*v* FITC, (**c**) 0.1% *w*/*v FITC*. Pig intestine stained with (**d**) 0.01% *w*/*v FITC* and (**e**) 0.1% *w*/*v FITC*. Scale bars: 100 µm.

To evaluate fluorescence visualization with the FBE, *FITC* diluted in deionized (DI) water was applied to tissue and pig-intestine samples at varying concentrations, as shown in Figure 7. The protocol included a control condition and two *FITC* concentrations: 0.01% *w*/*v* and 0.1% *w*/*v*. For tissue samples (Figure 7a–c), the control exhibited minimal fluorescence, whereas 0.01% *w*/*v FITC* produced moderate fluorescence with visible cellular features. At 0.1% *w*/v *FITC*, fluorescence intensity increased further, yielding brighter images. For pig-intestine samples (Figure 7d,e), 0.01% *w*/*v FITC* produced detectable fluorescence, whereas 0.1% *w*/*v FITC* yielded the strongest signal, corresponding to an approximately 2.5-fold increase in fluorescence intensity relative to Figure 7d. Together, these findings support the suitability of the FBE platform for fluorescence imaging and demonstrate that *FITC*-based contrast can be visualized with cellular-level detail under the tested experimental conditions.

### 4.2. Peptide Sequences and Candidate Selection

In this study, four *FITC*-labeled candidate peptides—SHS1*, SHS2*, FPP*, and CRL*—were evaluated as fluorescence probes in p16-overexpressing cervical cancer models. The peptide constructs and sequences are summarized in Table 2. Each peptide was synthesized with a C-terminal GGGSK linker conjugated to *FITC*, enabling fluorescence-based detection.

The four candidate probes were compared according to their peptide-associated fluorescence in the tested cell models. Among them, FPP*-FITC and CRL*-FITC showed the highest fluorescence-positive fractions in SiHa and CaSki cells and were therefore selected for further evaluation. In the present study, these findings are interpreted as evidence of preferential fluorescence enrichment in p16-overexpressing cervical cancer models, rather than as definitive proof of membrane-specific targeting or clinical diagnostic specificity. Accordingly, FPP*-FITC and CRL*-FITC were carried forward as the leading candidate probes for subsequent cell-based and ex vivo fluorescence-imaging experiments.

### 4.3. Peptide-Associated Fluorescence in Cervical Cancer Cell Lines

To evaluate peptide-associated fluorescence, the following cell lines were used: (1) A549 (human lung carcinoma; American Type Culture Collection [ATCC] *CCL-185*), (2) SiHa (human squamous cell carcinoma of the cervix; ATCC HTB-35), and (3) CaSki (Carcinoma of the Sloan-Kettering Institute; ATCC *CRL-1550*).

**Figure 8:**
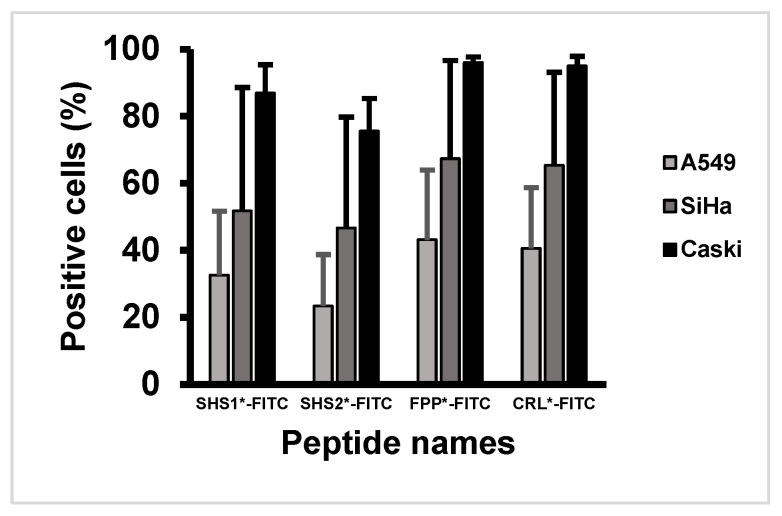
Percentage of fluorescence-positive cells after incubation with *FITC*-labeled peptides in A549, SiHa, and CaSki cell lines.

The resulting percentages of positive cells are summarized in Table 3 and Figure 8. In A549 cells, all peptides showed measurable fluorescence-positive fractions, with FPP*-FITC yielding 43.1 ± 20.8% positive cells and CRL*-FITC yielding 40.6 ± 18.1%. In SiHa cells, higher positive-cell fractions were observed, with FPP*-FITC showing the highest value at 67.3 ± 29.4%, followed by CRL*-FITC at 65.3 ± 27.8%. In CaSki cells, which showed the strongest signal among the tested models, FPP*-FITC and CRL*-FITC yielded 96.0 ± 1.7% and 95.0 ± 2.9% positive cells, respectively.

Overall, these data indicate preferential fluorescence enrichment of FPP*-FITC and CRL*-FITC in the p16-overexpressing cervical cancer cell lines relative to the A549 comparator line. However, because measurable fluorescence was also observed in A549 cells, the present results should not be interpreted as demonstrating absolute molecular specificity. In addition, because cells were incubated with the peptide prior to fixation, the assay reflects net peptide-associated fluorescence after short incubation. It may include contributions from surface association and/or cellular uptake.

### 4.4. FBE Imaging of FITC-Labeled Peptides and Ex Vivo Tissue-Array Samples

**Figure 9:**
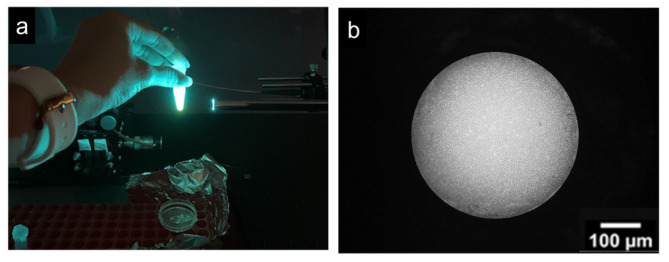
Fluorescence of CRL*-FITC peptide. (**a**) Emission spectrum (λ = 520 nm) visible under excitation (λ = 491 nm). (**b**) Fluorescence image captured with an FBE.

The fluorescence properties of the CRL*-FITC peptide are shown in Figure 9. Under excitation at 491 nm (10 mW), the peptide emitted visible fluorescence centered around 520 nm, which could be observed in an Eppendorf tube and captured using the FBE. These images confirm that the labeled peptide produced detectable fluorescence under the optical conditions used in this study.

**Figure 10:**
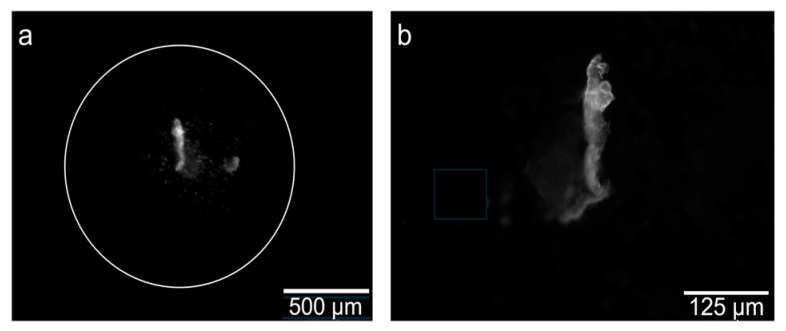
Fluorescence imaging of SiHa cells incubated with 50 µM CRL*-*FITC* peptide. (**a**) Image from the FBE. (**b**) Image from a standard fluorescence microscope.

Figure 10 compares fluorescence imaging of SiHa cells incubated with CRL*-FITC using two imaging modalities. Figure 10a shows the fluorescence image acquired using the FBE. In contrast, Figure 10b shows the corresponding fluorescence image acquired using a standard fluorescence microscope (MOTIC: PX43 FS6) with a 200 ms exposure and a 20× objective lens. The comparison demonstrates that the FBE was able to visualize peptide-associated fluorescence patterns in the cell sample under the tested conditions.

**Figure 11:**
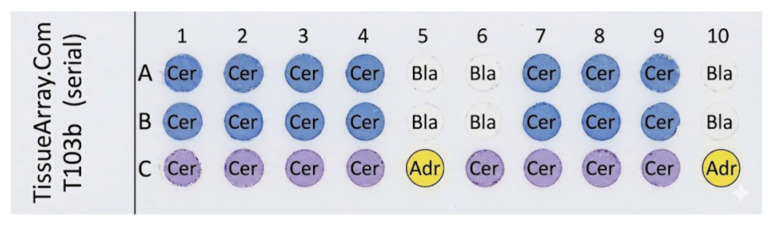
Layout of human tissue array from TissueArray.com, including cervical cancer (A, B; dark blue circles), normal cervical (C; purple circles), and adrenal tissues (C5, C10; yellow circles), with blank samples (A5, A10, B5, B10; white circles).

**Figure 12:**
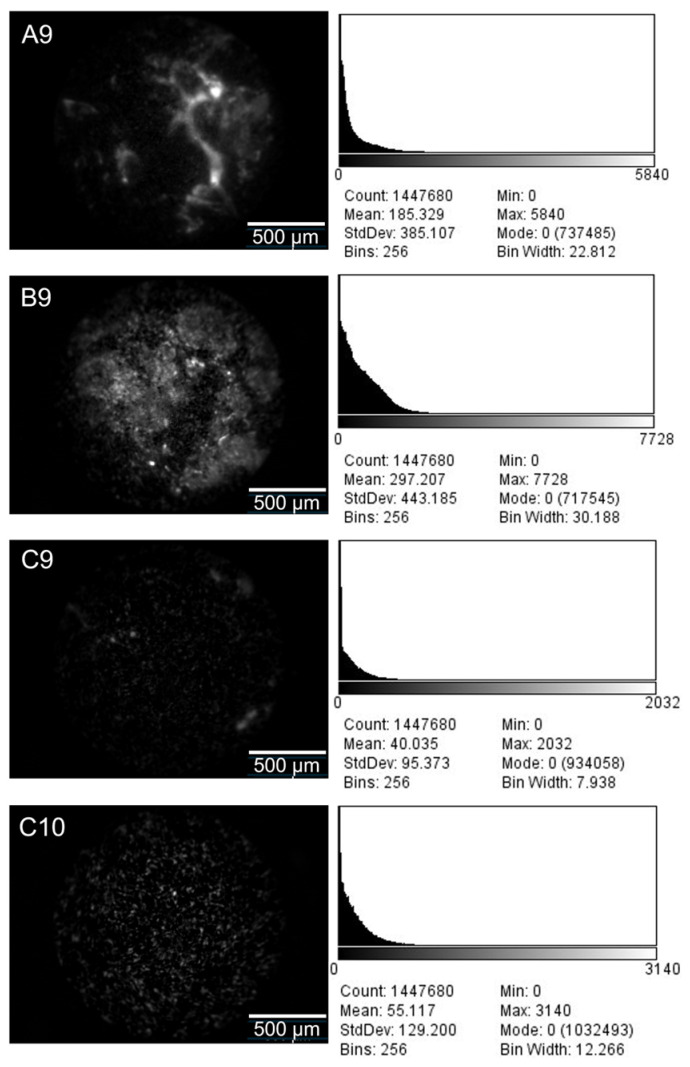
Fluorescence images and intensity histograms of tissue samples. Mean fluorescence intensities for A9, B9, C9, and C10 were 185.33, 297.21, 40.04, and 55.12, respectively, at CRL*-FITC concentration of 25 µM.

The layout of the commercial human tissue array used in this study is shown in Figure 11. The array included cervical cancer tissues (A and B), normal cervical tissues (C), adrenal tissues (C5 and C10), and blank samples (A5, A10, B5, and B10). CRL*-FITC was evaluated on this array at a peptide concentration of 25 μM. Figure 12 presents representative fluorescence images and corresponding intensity histograms for selected cervical cancer, normal cervical, and adrenal tissue samples.

Quantitative analysis of the image histograms in Figure 12 yielded mean fluorescence intensities of 185.33 ± 385.11 (A9) and 297.21 ± 443.19 (B9) for the malignant tissue samples, compared with 40.04 ± 95.37 (C9) for normal cervical tissue and 55.12 ± 129.20 (C10) for adrenal tissue. The large standard deviations reflect substantial heterogeneity in the underlying pixel-intensity distributions, consistent with punctate fluorescence signal over a relatively dark background. Relative to the normal cervical reference, this corresponds to an approximately 4.6- to 7.4-fold increase in mean fluorescence intensity in the malignant samples. Statistical comparison of the pixel-intensity distributions showed a significant difference between the malignant and non-malignant samples (*p* < 0.001).

These findings indicate that CRL*-FITC generated a higher fluorescence signal in the malignant ex vivo tissue-array samples than in the non-malignant comparator tissues under the tested conditions. However, given the wide dispersion of signal intensities and the limitations of archived ex vivo tissue-array material, these data should be interpreted as exploratory proof-of-principle evidence of optical contrast generation, rather than definitive evidence of clinical diagnostic performance.

### 4.5. Telehealth Platform for Remote Consultation

**Figure 13:**
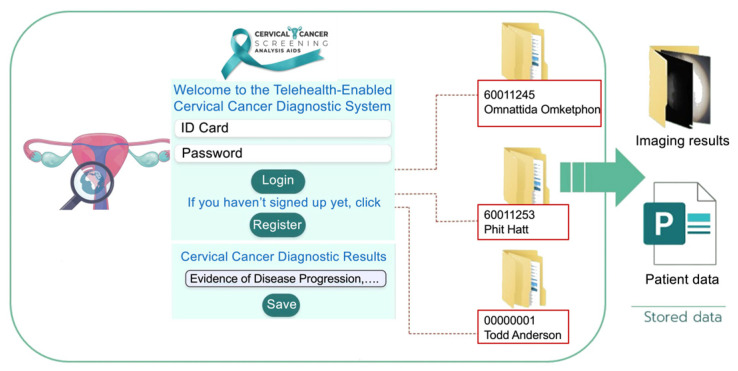
Telehealth platform interface for remote consultation, case entry, and image management.

The telehealth platform developed in this study is shown in Figure 13. The figure illustrates the platform’s registration and login interface, which provides access control for authorized users. The platform includes modules for case registration, remote consultation, structured diagnostic data entry, and image storage and retrieval. These functions were designed to support the organization and communication of imaging data within the proposed telehealth workflow.

Figure 13 also shows the platform’s core user interfaces. A conferencing interface supports remote communication between an operator at the image-acquisition site and a specialist at a central site. A structured reporting form allows findings to be entered and saved to a local or cloud-based database. In addition, a folder view organizes stored diagnostic images for later review and case documentation. Together, these interfaces demonstrate the technical integration of remote consultation, secure documentation, and imaging data retrieval within the proposed platform.

**Figure 14:**
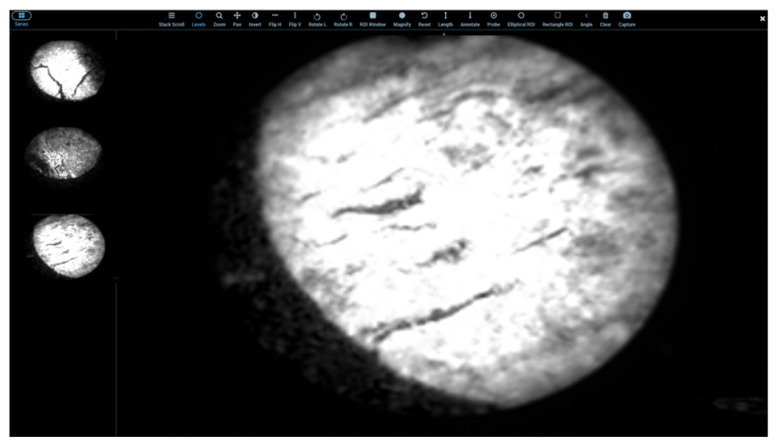
Representative endoscopic images displayed in the digital viewer interface.

Figure 14 presents representative images displayed in the platform’s digital viewer. The left panel shows three sequential endoscopic image frames, whereas the right panel shows a magnified view of a selected frame. The viewer includes zoom, pan, inversion, annotation, and calibrated measurement tools to support visual inspection and image review. In the present study, the telehealth platform should be interpreted as a technical remote consultation interface rather than a clinically validated telemedicine diagnostic platform.

### 4.6. System Performance and Security Validation

To assess the technical feasibility of remote consultation, we evaluated both data security and transmission latency. All information exchanged between the remote site and the specialized center was secured using Transport Layer Security (TLS) 1.3 [28] to protect data in transit. Static patient records and diagnostic images stored in the SQL database were encrypted using Advanced Encryption Standard (AES)-256 [29] to strengthen data protection against unauthorized access.

Operational latency is an important parameter in telemicroscopy and remote image review. A simulated remote consultation workflow was established using the 5.0-megapixel FLIR Grasshopper2 camera over standard 4 G/5 G network conditions. Under these test conditions, the telehealth platform demonstrated an average end-to-end latency of <500 ms. In addition, image transmission used lossless compression to preserve image detail during streaming. This latency threshold is sufficient to maintain effective remote guidance and verbal coordination between the local endoscopist and the remote specialist. These findings support the technical feasibility of secure, low-latency remote image visualization within the proposed platform. However, they do not establish the accuracy of remote diagnosis, interobserver agreement, or clinical workflow performance.

## 5. Discussion

This study presents an integrated platform that combines FBE, *FITC*-labeled candidate peptides, and a telehealth interface for remote consultation. The principal contribution of the work is the preclinical feasibility of combining these optical, molecular, and digital components into a single workflow. The FBE system provided cellular-level fluorescence imaging in a portable format, the peptide experiments showed preferential fluorescence enrichment in p16-overexpressing cervical cancer models, and the telehealth platform supported secure data transmission with low-latency remote visualization. Taken together, these findings support the engineering integration of the proposed platform.

The optical component of the platform provides a practical basis for portable fluorescence imaging. The reported field of view and lateral resolution indicate that the FBE platform can visualize fluorescence patterns at the cellular scale, which is relevant for lesion-directed imaging applications. At the same time, the current optical design is based on wide-field epi-fluorescence imaging and does not provide optical sectioning. As a result, fluorescence originating from superficial and subsurface structures may be integrated into the same image, potentially reducing contrast in thicker tissues and limiting depth discrimination. This limitation reflects an intentional engineering trade-off. Fiber-based confocal imaging systems can achieve substantially better optical sectioning but generally require more complex scanning mechanisms, tighter optical alignment, and higher system costs, and often provide a much smaller field of view. Such constraints may reduce practicality for rapid survey imaging and field deployment. In contrast, the present wide-field FBE configuration was selected because it offers a simpler, more compact, and more portable architecture with a larger field of view, which is advantageous for point-of-care and telehealth-oriented applications. However, this comes at the expense of optical sectioning and depth discrimination. This limitation is particularly relevant in the context of cervical neoplasia, where accurate localization of an abnormal signal within the epithelial layers may be important for translational interpretation. Accordingly, the current optical results should be interpreted as demonstrating fluorescence imaging capability rather than as definitive layer-specific lesion assessment.

The molecular component of the platform was motivated by the use of p16 as a biomarker associated with HPV-related cervical neoplasia. In the present study, FPP*-FITC and CRL*-FITC showed higher fluorescence-positive fractions in SiHa and CaSki cells than in the A549 comparator line, and CRL*-FITC generated a higher fluorescence signal in malignant ex vivo tissue-array samples than in the non-malignant comparator tissues under the tested conditions. These findings support the use of peptide-based fluorescence probes to generate biologically relevant optical contrast. However, the results should be interpreted cautiously. Because p16 is predominantly an intracellular biomarker, we hypothesize that the hydrophobic *FITC* fluorophore and the positively charged lysine in the GGGSK linker may facilitate initial membrane interaction and subsequent cellular internalization, allowing the probes to access and accumulate at intracellular p16 targets. This requirement for intracellular access has direct implications for the translational feasibility of *in vivo* optical targeting. Specifically, future clinical workflows would likely require topical application of the probe followed by a dedicated incubation period to permit sufficient membrane permeation and intracellular accumulation prior to endomicroscopic imaging, rather than instantaneous visualization. While this is a common workflow for topical contrast agents, the current experiments do not definitively establish the relative contributions of surface binding versus intracellular accumulation, and future studies will be needed to precisely map the internalization kinetics. In addition, the live-cell flow-cytometry protocol measured peptide-associated fluorescence after a short incubation period and therefore reflects net fluorescence enrichment rather than direct evidence of membrane-specific binding.

Our prior phage-display study provides useful background for part of the present peptide strategy [18]. In addition, CRL* was adapted from a previously reported HPV-transformed-cell targeting peptide identified by Robinson et al. [19]. In that earlier work, candidate p16-binding peptides were identified by phage-display selection, showed preferential interaction with p16-overexpressing models, and one leading candidate demonstrated competitive reduction in anti-p16 antibody staining, supporting the plausibility of p16-related binding. Nevertheless, that earlier work also concluded that additional inhibitory, biophysical, and structural studies would still be required to define the precise binding epitope and mechanism. For this reason, the present manuscript should cite that prior study as mechanistic support for the peptide rationale, but not as a substitute for direct mechanistic validation of the current *FITC*-labeled synthetic peptides.

The cell-line results also indicate that the present probes do not demonstrate absolute molecular specificity. In particular, the measurable fluorescence-positive fractions observed in A549 cells suggest that background binding and/or nonspecific uptake were present under the assay conditions used here. A plausible explanation is that the observed background signal reflects, at least in part, nonspecific membrane interactions and/or endocytic uptake influenced by the physicochemical properties of the labeled probes.

Fluorophore labeling has been shown to alter peptide–membrane interactions and cellular distribution, and the cationic GGGSK linker may also contribute to electrostatic interactions with negatively charged cell surfaces. To mitigate these effects and improve selectivity in future translational stages, probe optimization must focus on modifying these physicochemical properties. Potential strategies include substituting *FITC* with more hydrophilic fluorophores, neutralizing the linker charge to reduce nonspecific membrane interactions, or employing multivalent peptide presentations to enhance specific target avidity over nonspecific endocytic uptake. However, the present study was not designed to directly resolve these mechanisms.

Therefore, the current data are more appropriately described as showing preferential fluorescence enrichment in p16-overexpressing cervical cancer models than as proving strong or exclusive specificity. This distinction is important for translational interpretation because p16 overexpression is not exclusive to cervical disease and may also be encountered in other gynecologic malignancies and HPV-related lower genital tract lesions, including uterine serous carcinoma, subsets of ovarian carcinoma, and high-grade vulvar intraepithelial neoplasia. Accordingly, p16 alone is an imperfect surrogate biomarker for future screening-oriented applications. In future translational development, it may therefore be useful to consider multi-marker strategies, such as approaches incorporating p16/Ki-67 dual-stain triage, and to integrate them into established colposcopic and histopathologic workflows. The ex vivo tissue-array experiment provides preliminary evidence that CRL*-FITC can generate a higher fluorescence signal in malignant cervical specimens than in comparator tissues. However, this part of the study remains exploratory. The tissue-array data showed wide dispersion, with standard deviations exceeding the means in some samples, indicating substantial heterogeneity and limiting the strength of inference regarding reproducibility. In addition, archived commercial tissue-array material does not replicate the living cervical surface. Such specimens may not preserve native epithelial barrier properties and do not reproduce clinically relevant factors such as mucus, bleeding, inflammation, lesion topography, or dynamic probe distribution. Consequently, the present ex vivo findings do not address peptide penetration through cervical mucus, performance in inflammatory cervicitis, discrimination among CIN grades, or false-positive rates in realistic clinical settings. The use of adrenal tissue should likewise be interpreted only as an additional non-cervical exploratory control available on the commercial array. In contrast, normal cervical tissue remains the more relevant biological comparator.

The telehealth component of the platform addresses an important practical problem, namely the difficulty of obtaining timely specialist input in remote or resource-limited settings. In this study, the software architecture demonstrated secure user authentication, structured case documentation, image and video exchange, and end-to-end latency of less than 500 ms under simulated network conditions. These results support the technical feasibility of remote image review within the proposed workflow. However, they should not be interpreted as clinical validation of telemedicine performance. No interobserver agreement study, remote diagnostic concordance analysis, usability assessment, or interoperability evaluation was performed. Therefore, the telehealth results should be presented as proof of technical integration, not as evidence that the platform has already improved clinical decision-making or workflow efficiency in real-world practice.

Overall, the present work is best understood as a preclinical feasibility study of an integrated FBE–peptide–telehealth platform for future cervical imaging applications. Its main value lies in demonstrating that these three components can be combined into a technically coherent system capable of generating peptide-associated fluorescence contrast, visualizing that contrast with a portable optical device, and transmitting the resulting data through a secure telehealth framework. Further mechanistic studies, more rigorous specimen-based validation, and prospective clinical investigation will be required before the platform’s diagnostic performance and screening utility can be established.

### Study Limitations

Several limitations should be acknowledged. First, the study was entirely preclinical and did not include *in vivo* cervical imaging, prospective patient recruitment, histopathologic correlation, CIN stratification, or clinical performance analysis such as sensitivity, specificity, or ROC assessment. Second, the peptide mechanism was not directly resolved; thus, the current data do not distinguish between specific binding, internalization, and nonspecific uptake. In addition, because p16 overexpression is not exclusive to cervical neoplasia and may also occur in other gynecologic malignancies or HPV-related lesions, the specificity of any future clinical application would need to be evaluated carefully in broader comparative settings. Third, the ex vivo tissue-array analysis was based on archived commercial material with limited physiologic relevance to the living cervical surface and substantial signal variability. Fourth, the wide-field FBE system lacks optical sectioning, which may reduce contrast and depth discrimination in more complex tissues. Fifth, the telehealth evaluation was limited to technical testing of data security and latency in a simulated environment and did not include clinical usability or diagnostic concordance validation. Finally, some experiments would benefit from more explicit reporting of sample numbers, replicate structure, and statistical procedures. These limitations should be considered when interpreting the present findings and when positioning the study relative to established cervical screening methods.

Despite these limitations, the study provides a useful foundation for future translational development by establishing the feasibility of combining portable fluorescence imaging, peptide-based molecular contrast, and telehealth-enabled remote consultation in a single platform. The next phase of work should focus on clarifying peptide-target interaction, improving biological specificity, expanding specimen-based validation, and evaluating the platform in near-clinical and clinical settings.

## 6. Conclusions

This study presents an integrated platform that combines FBE, *FITC*-labeled candidate peptides, and a telehealth interface for remote consultation. The FBE system achieved a field of view of 1700 µm and a lateral resolution of 4 µm, enabling cellular-level fluorescence imaging in a compact, portable format. Among the tested probes, FPP*-FITC and CRL*-FITC showed higher peptide-associated fluorescence in the p16-overexpressing cervical cancer cell lines than in the A549 comparator line. In comparison, CRL*-FITC generated a higher fluorescence signal in malignant ex vivo tissue-array samples than in the non-malignant comparator tissues under the tested conditions. In parallel, the telehealth platform supported secure data handling and low-latency remote visualization in a simulated consultation workflow.

Taken together, these findings support the technical and preclinical feasibility of integrating targeted fluorescence imaging, portable fiber-bundle endomicroscopy, and telehealth into a single platform. However, the present work should not be interpreted as establishing clinical screening performance or immediate deployment within cervical cancer screening programs. The biological evaluation remains limited to established cell lines and archived ex vivo tissue-array material, and the telehealth component was assessed only at the technical level rather than through diagnostic concordance or clinical usability studies. In addition, the current data do not establish absolute molecular specificity or resolve the mechanism of peptide interaction with p16-overexpressing cells.

Accordingly, this study is best understood as a preclinical feasibility evaluation of an integrated optical, molecular, and digital workflow for future cervical imaging applications. Further work should focus on mechanistic validation of peptide targeting, broader specimen-based evaluation with clearer statistical characterization, and prospective *in vivo* or near-clinical studies with histopathologic correlation before the platform’s diagnostic utility in cervical screening can be determined.

## Figures and Tables

**Table 1:** List of optical system components and their specifications.

Label	Part Number	Description
L1	AC254-100-B	f = 100.0 mm, Ø1” achromatic doublet, AR Coated: 650–1050 nm
L2	AC254-150-A	f = 150.0 mm, Ø1” achromatic doublet, AR Coated: 400–700 nm
L3	MPLN10×	objective lens 10×, working distance: 10.6 mm, 0.25 NA
L4	ACL2520U	f = 20.1 mm, Ø25” aspheric condenser, Uncoated, 0.6 NA
EX1	FF02-482/18-25	single band exciter, λ_max_ > 93% 473–491 nm, center-wavelength: 482 nm
EM1	FF02-524/24-25	single band emitter, center-wavelength: 524 nm, bandwidth: 24 nm
DM1	Di03-R488-t1	single band dichroic, λ_max_ > 93%, bandwidth: 499.8–1200 nm
M1	NB1-J11	Ø1” Kr-Ion Laser Mirror, 520–647 nm
F1	M92L02	multimode fiber, diameter: 200 µm, 0.2 NA
FB	FIGH-30-850N	fiber diameter: 650 µm, number of optical fibers = 30,000
GL	GT-IFRL-100-005-50-CC	lens diameter: 1.0 mm, working distance: 5 mm, 0.50 NA

**Table 2:** Candidate peptide sequences used in the present study, derived from prior phage-display peptide studies relevant to cervical cancer targeting [18,19].

Peptide ID	Parent Peptide in Prior Study	Amino-Acid Sequence	C-Terminal Linker/Labeling Format	Use in Present Study
SHS1*	pep 1 [18]	Ser-His-Ser-Leu-Leu-His-His	GGGSK-*FITC*	Screened in cell-based assay
SHS2*	pep 2 [18]	Ser-His-Ser-Leu-Leu-Ser-Ser	GGGSK-*FITC*	Screened in cell-based assay
FPP*	pep 5 [18]	Phe-Pro-Pro-Ser-Val-Ile-Arg	GGGSK-*FITC*	Selected for further ex vivo evaluation
CRL*	clone 1 [19]	Cys-Arg-Leu-Thr-Gly-Gly-Lys-Gly-Val-Gly-Cys	GGGSK-*FITC*	Selected for further ex vivo evaluation

**Table 3:** Percentage of fluorescence-positive cells after incubation with *FITC*-labeled peptides.

Peptide	Positive Cells (%)		
	A549 Lung Cancer	SiHa Cervical Cancer	CaSki Cervical Cancer
SHS1*-FITC	32.6 ± 19.1	51.7 ± 36.9	86.9 ± 8.5
SHS2*-FITC	23.4 ± 15.4	46.7 ± 33.1	75.5 ± 9.8
FPP*-FITC	43.1 ± 20.8	67.3 ± 29.4	96.0 ± 1.7
CRL*-FITC	40.6 ± 18.1	65.3 ± 27.8	95.0 ± 2.9

## Data Availability

De-identified data products that support the main findings of this work, including fluorescence imaging from commercial human tissue arrays (TissueArray.com) and established cell lines, are available to accredited scientific researchers from the corresponding author upon reasonable request. All requests will be considered in cooperation with the Institutional Review Board of King Mongkut’s Institute of Technology Ladkrabang (Protocol: EC-KMITL 64 034) and may require a formal data-use agreement. Within these regulatory and ethical constraints, we actively encourage collaboration and will make every effort to facilitate data access for bona fide academic research.

## Abbreviations

The following abbreviations are used in this manuscript:

VIA	Visual Inspection with Acetic Acid
VI	Visual Inspection
SVA	Single Visit Approach
Pap	Papanicolaou
HPV	Human Papillomavirus
DNA	Deoxyribonucleic Acid
ACOG	American College of Obstetricians and Gynecologists
USPSTF	United States Preventive Services Task Force
FBE	Fiber Bundle Endomicroscopy
FITC	Fluorescein Isothiocyanate
EMS	Emergency Medical Services
HIE	Health Information Exchange
ID	Identification
UI	User Interface
HTML	Hypertext Markup Language
PHP	Hypertext Preprocessor
OTP	One-Time Password
API	Application Programming Interface
A549	Human lung carcinoma
ATCC	American Type Culture Collection
SiHa	Human squamous cell carcinoma of the cervix, overexpressing p16
CaSki	Carcinoma of the Sloan-Kettering Institute (a human cancer cell line derived from a squamous cell carcinoma of the cervix)
BSA	Bovine Serum Albumin
TLS	Transport Layer Security
AES	Advanced Encryption Standard
SQL	Structured Query Language
FOV	Field of View
CMOS	Complementary Metal-Oxide-Semiconductor
CCD	Charge-Coupled Device
GRIN	Gradient Index
NA	Numerical Aperture
WD	Working Distance
SMA	Sub-miniature version A
AR	Anti-Reflective
PBS	Phosphate-Buffered Saline
DI	Deionized
RTCOG	Royal Thai College of Obstetricians and Gynaecologists
